# Gastric ultrasound: Enhancing preoperative risk assessment and patient safety

**DOI:** 10.1177/17504589241302220

**Published:** 2024-12-11

**Authors:** Luke Kar Man Chan

**Affiliations:** 1 Concord Repatriation General Hospital, Sydney, NSW, Australia; 2 Faculty of Medicine and Health, The University of Sydney, Sydney, NSW, Australia; 3 School of Medicine and Dentistry, Griffith University, Southport, QLD, Australia

**Keywords:** Anaesthetics–management and care, Bariatric surgery, Clinical investigations, Endoscopy, Evidence-based practice, Pre-assessment, Risk management, Clinical education and training

## Abstract

Perioperative pulmonary aspiration is a critical complication linked to significant morbidity and mortality, particularly in high-risk populations such as patients with diabetes, obesity, gastroparesis, or those using Glucagon-Like-Peptide-1 receptor agonists (GLP-1 RAs). Standard fasting protocols may not be appropriate for these patients, as they have increased propensity of delayed gastric emptying, hence increasing the complex of the preoperative risk assessment. Gastric ultrasound (GUS) provides a non-invasive, reliable method for assessing gastric content and volume, enabling anaesthesia professionals to make informed decisions regarding aspiration risk, airway management, and surgical scheduling. By identifying patients with elevated gastric volumes, GUS has the potential to reduce aspiration-related complications and unnecessary surgical cancellations.

Despite its clear clinical benefits, the adoption of GUS in anaesthetic practice remains limited, primarily due to the technical skill required for accurate quantitative assessments. Qualitative evaluations of gastric contents are simpler for beginners, but precise volume measurements, essential for risk stratification, demand more extensive training. Recent studies demonstrate that with structured training, even novice operators can achieve high diagnostic accuracy. Artificial intelligence (AI) can further enhance GUS utility by automating volume calculations, guiding probe placement, and providing real-time feedback. These capabilities could significantly shorten the learning curve and improve consistency in risk assessment.

Incorporating GUS and AI tools into anaesthesia training can overcome adoption barriers, enabling clinicians to more accurately assess aspiration risk and enhance patient safety in perioperative care.

## Perioperative aspiration risk and gastric ultrasound

Perioperative pulmonary aspiration is a serious complication associated with significant morbidity and mortality (Kozlow et al 2003). Patients who develop aspiration pneumonia face a fourfold increase in the risk of admission to intensive care units and a 7.6-fold increase in hospital mortality (Kozlow et al 2003). Preoperative fasting can help reduce aspiration risk, but there is a lack of clear guidance on appropriate fasting periods for high-risk patients, including those with diabetes, gastroesophageal reflux disease, obesity, pregnancy or opioid use (Rüggeberg et al 2024). Alarmingly, the incidence of gastroparesis and the prevalence of diabetes and obesity are rising (Ong et al 2023, Phelps et al 2024, Ye et al 2021). Recently, the increasing use of Glucagon-Like-Peptide-1 receptor agonists (GLP-1 RAs) for diabetes and weight loss has raised anaesthetic safety concerns (Chan 2024). Case reports and observational studies suggest that patients on GLP-1 RAs have a higher likelihood of retaining gastric contents even after following standard fasting protocols (Martis et al 2024). Moreover, anaesthesia professionals frequently encounter the challenge of patients with unclear fasting status, particularly those with cognitive impairment, language barriers or compliance issues. With the rise in older surgical patients, the prevalence of cognitive impairment is also increasing, making it increasingly challenging to obtain accurate fasting histories (Chan et al 2024, Partridge et al 2018).

Gastric ultrasound (GUS) offers anaesthesia professionals a non-invasive, effective method to assess gastric content and volume, guiding aspiration risk assessment and decisions on airway management and surgical timing. For elective procedures, the presence of solids, thick liquids, or clear fluids exceeding 1.5 mL/kg often warrants cancellation or postponement of surgery (Perlas et al 2013). Perlas’ model demonstrates a strong correlation between cross-sectional area and gastric volume in the right lateral position (Zhang et al 2020). Increased utilisation of GUS could be pivotal for preventing aspiration-related complications while avoiding unnecessary surgery cancellations, leading to improved cost-effectiveness in perioperative care. Despite its clear advantages, GUS remains underutilised and is still not widely integrated in anaesthesia training programmes, including in the United Kingdom and Australia.

## Challenges and limitations of GUS training

A key barrier to the widespread adoption of GUS in anaesthesia is the learning curve associated with accurate volume assessment. While qualitative assessments of gastric contents are easier to learn (Bouvet et al 2023), quantitative evaluations, which require measuring the cross-sectional area of the stomach, demand more expertise and experience (Tankul et al 2022). However, these quantitative assessments are crucial to provide precise risk stratification, as low residual gastric volume is often normal in fasted patients with minimal aspiration risk (Van de Putte & Perlas 2014). Novice operators may struggle with interpretation, resulting in unnecessary cancellations from false positives or aspiration events from false negatives.

However, these challenges are surmountable. A recent study demonstrated that anaesthetists with limited GUS experience can achieve high diagnostic accuracy with adequate training (Tankul et al 2022). Integrating GUS into anaesthesia curricula requires a commitment to structured, hands-on training, but long-term benefits such as improved patient safety and fewer cancelled cases could outweigh the initial resource investment.

## The role of artificial intelligence in enhancing GUS training and implementation

Artificial intelligence (AI) offers potential to overcome the challenges of GUS training. AI algorithms, particularly machine learning, can assist operators by providing real-time feedback on probe placement and automating volume calculations, thereby reducing operator dependency and improving accuracy (Ferraz et al 2023). Furthermore, AI systems can improve their performance through additional data and feedback (Krittanawong et al 2023).

To my knowledge, Jin et al (2023) is the only study to date which tests AI-based gastric volume estimation. This large study evaluates the prediction of a ‘full stomach’ in patients undergoing gastrointestinal endoscopy. GUS ML models, with an area under the receiver operating characteristic curve (AUROC) of 0.903 outperformed the clinical data models (AUROC of 0.829) and the point-of-care ultrasound-based linear model proposed by Bouvet et al (2011) (AUROC of 0.84). Future research should explore integrated AI systems for performing real-time GUS analysis, as Jin et al (2023) focused exclusively on retrospective AI analysis. This approach has shown promise in other applications, such as point-of-care echocardiography (Mika et al 2024). As AI technology advances, integrating AI tools into GUS could shorten the learning curve for anaesthesia professionals.

## Conclusion

Given the increasing complexity of perioperative patient populations and the evolving aspiration risks, incorporating GUS into anaesthesia training curricula should be considered. This would empower anaesthesia professionals to provide safer, more personalised care. The future integration of AI could further enhance GUS utility, making it easier to adopt and implement effectively.
